# Editorial: What's New in Endocrinology?

**DOI:** 10.3389/fendo.2019.00838

**Published:** 2019-12-03

**Authors:** Jeff M. P. Holly, Derek LeRoith

**Affiliations:** ^1^IGFs and Metabolic Endocrinology Group, Translational Health Sciences, Faculty of Health Sciences, Bristol Medical School, University of Bristol, Bristol, United Kingdom; ^2^Endocrinology, Diabetes and Bone Diseases, Icahn School of Medicine at Mount Sinai, New York, NY, United States

**Keywords:** obesity, reproduction, stress, cancer, aging

As it approaches a decade since Frontiers in Endocrinology was launched, the Chief Editors have commissioned a series of articles to reflect the continuing dynamic evolution of the science at the Frontiers of Endocrinology. These articles highlight recent breakthroughs or advances, new technologies, or challenges in the field of endocrinology. As with any dynamic field the frontiers are ever changing and these articles exemplify some of the recent developments together with some of the new questions and challenges for the future. The articles cover many different areas of endocrinology including issues involved in some of the biggest health challenges facing today's society such as stress, obesity, reproduction, cancer, and aging.

The greatest challenge to health provision in virtually every country across the globe is obesity; the scale of the epidemic threatens to outstrip resources in even the richest of societies (1). The burden of morbidity arising from obesity and its sequelle of type 2 diabetes, cardiovascular disease, and cancer present many challenges that are not restricted to those providing healthcare but reaching across society. Big questions of how we feed ourselves and how we live our lives need to be addressed (2); but some of the most fundamental questions center on how the body regulates energy balance: essentially an endocrine question. Obesity is the direct result of a chronic imbalance between energy intake and energy expenditure with the excess energy stored in adipose tissue. Adipose tissue was traditionally considered a relatively inert tissue comprising cells that just functioned as stores of excess energy in the form of lipids. This changed during the 1990's when it was discovered that adipocytes were an important source of key hormones such as leptin and adiponectin with important roles in regulating both energy intake and expenditure (3). Interest in adipose tissue was enhanced further around a decade ago when imaging techniques using labeled glucose revealed that humans possess brown adipose tissue (BAT) (4). Whereas, white adipocytes contain large stores of fats, in contrast brown adipocytes contain large numbers of mitochondia in which uncoupling protein 1 (UCP1) enables energy to be dissipated as heat rather than being stored. Until then it was considered that brown adipocytes were an important site of thermogenesis and hence energy expenditure in rodents but were considered absent from humans. This raised the potential of a new strategy to address human obesity by targeting BAT to enhance energy expenditure. In a comprehensive article Carpentier et al. review the challenges that have been encountered in the subsequent decade of research into BAT in humans. They address key questions such as the true extent of BAT in humans; whether the original imaging techniques underestimated the total mass of BAT and to what extent “beiging” of white adipocytes, with the induction of UCP1, can occur in humans. These are critical questions that could establish whether beige and brown adipocytes in humans could be an effective target to bring about changes in energy expenditure that are of therapeutic benefit.

In addition to the advances in our understanding of energy expenditure there have been major progress in our knowledge of the endocrine controls of energy intake via hormones that control hunger and satiety and hence determine food intake (5). Life within modern societies is increasingly stressful and whether this impacts on the controls of appetite and satiety resulting in over-eating is an interesting question. The effects of stress on the gut-brain signaling pathways and the neuropeptides involved are reviewed by Stengel and Taché.

The management of obesity remains a huge health challenge. Numerous dietary and lifestyle changes have been proposed and all have achieved modest weight loss that is invariably soon regained. The pharmaceutical industry has invested considerably in developing medications to treat the huge potential market with a long trail of failures with many concerns regarding adverse effects and long-term safety (2). While nutritionists, physicians, and pharmacologists have floundered in the quest to control the obesity epidemic; surgeons have developed a number of bariatric procedures that result in effective and sustained weight loss. One of the surprising observed effects of bariatric surgery has been the remarkable correction of the metabolic disturbances often resulting in complete remission of type 2 diabetes. That this effect is often apparent within days of surgery and before any appreciable weight loss has challenged many of our dogmas of the links between obesity and its associated metabolic disturbances. Both Ma and Vella and Laferrère and Pattou have reviewed the potential new insights into metabolic endocrinology that these observations may provide. The clues from the anatomical differences between the different surgical procedures are reviewed by Ma and Vella. They describe how such anatomical distinctions can provide insights into the various hormonal pathways and in particular the interactions between regions of the gut and the pancreas. They also touch on other interesting, less well-appreciated effects, such as how the surgery and endocrine changes can alter the perception of sweet-taste and hence alter calorie intake. The most studied surgical intervention is Roux-enY gastric bypass and Laferrère and Pattou review these studies with an emphasis on what these studies have revealed regarding the gut endocrine system. They highlight the many new questions raised in relation to the role of satiety hormones, incretins, and bile acids. Our concepts of bile acids have been transformed from being regarded as just soaps that aid in the uptake of dietary fats to being a previously unappreciated complex endocrine system.

While the obesity epidemic has added to the focus on type 2 diabetes it has also become clear that there has been a 3% annual increase in the incidence of type 1 diabetes and Jacobsen et al. provide an overview of the development of strategies to prevent this and avoid the need for lifelong treatment. In order to prevent type I diabetes it is important to understand the natural history of the development of the autoimmunity that results in pancreatic beta cell destruction and the onset of type 1 diabetes. The challenges of studying populations prior to the disease onset and how this is being addressed around the world are described. These studies have informed the various trials for primary prevention in subjects at risk and secondary prevention in those already exhibiting evidence of autoimmunity. To date these studies have had limited success and new and future strategies are discussed.

In a fine exposition of how studying one component can help inform on how inter-connected the endocrine system has become; Eiden and Jiang review how new observations of adrenal chromaffin cells have contributed to our understanding of how we coordinate response to stress. The adrenal medulla has conventionally been considered the source of epinephrine to coordinate the cardiovascular, neuronal, and metabolic responses to stress. In this overview they describe recent observations of sympathetic nervous system regulation of chromaffin cell function and its secretion of not just epinephrine but also a rich cocktail of novel bioactive peptides. This new evidence is synthesized into a broader understanding of how metabolic, cardiovascular, and inflammatory responses are integrated. They also highlight interesting new questions that have arisen from this work; such as whether the sensory nervous system and immune/inflammatory systems are looped-in together via the adrenal medullary stress response and what are the broader endocrine functions of the many bioactive peptides secreted from the chromaffin cells.

Population control and reproductive health remain major health issues globally. The important role of androgens both in ovarian follicle selection to ensure mono-follicular ovulation in women and in the normal cyclical secretion of estrodiol is reviewed by Franks and Hardy. They focus on recent advances regarding the role of androgens in the development of polycystic ovary syndrome (PCOS), which remains the most common endocrine disorder in women of reproductive age.

One of the recent advances in techniques for maintaining fertility in women has been ovarian tissue cryopreservation (OTC) and transplantation. Originally developed to assist prepubertal girls and young women faced with reductions in the ovarian reserve due to pathologies, such as malignancies, or due to aggressive therapies that damage the ovary, Kristensen and Andersen discuss the many issues and challenges with extending this technique to more broader applications. Using this technique to restore fertility to women with anovulatory PCOS is discussed. The many issues surrounding the more controversial application of the technique to enable healthy women to postpone childbearing into their more advanced years is also addressed.

During pregnancy a woman's endocrine system kicks into overdrive with most hormones adapting to enable the mother to meet the additional metabolic demands and to provide an optimal environment in which the fetus can develop. Among all of these hormonal changes vitamin D plays an under-appreciated role both in ensuring adequate calcium availability for fetal bone development and in enhancing maternal tolerance to the presence of paternal and fetal alloantigens. Recent advances in our understanding of the part played by vitamin D-binding protein (VDBP) in facilitating these roles is reviewed by Karras et al.. The ongoing questions regarding the role of VDBP in important clinical issues such as preeclampsia, preterm birth, and gestational diabetes are discussed.

Neuropeptide G protein-coupled receptors (GPCRs) are over-expressed in many different cancers; not just the relatively rare neuroendocrine tumors but also in some common cancers such as small cell lung cancers. The potential targeting of specific GPCRs for the development of novel cancer therapies is reviewed by Moody et al.. The development of agents that target receptors for bombesin, neurotensin, vasoactive intestinal peptide, and somatostatin are described in relation to the detection and treatment of both endocrine and non-endocrine cancers.

Recent advances in the genetics, biochemical characterization, and imaging of pheochromocytomas (PCCs) and paragangliomas (PGLs) are reviewed by Alrezk et al.. These are challenging cancers to treat and although most can be cured by surgery on rare occasions they metastasize and for these there are currently no approved treatments. The potential of systemic therapies, that have largely been developed to treat other cancers, is reviewed by Jimenez. Different strategies that have been designed to target each of the accepted “hallmarks” are discussed in relation to their application to treating PCCs and PGLs. These include different strategies to inhibit angiogenesis, cell proliferation, invasion, and metastasis, to enhance the induction of cell death and the recently developed immunotherapies.

As more and more people survive into advanced ages the problems of the elderly become an increasing burden on clinical services. The prevalence of thyroid nodules in people over the age of 60 years is extremely high (50–70%) and although most of these are benign (85–95%) it is important to distinguish the few that can become malignant and require surgery. The current status of molecular markers for the differential diagnosis of malignant, vs. benign, thyroid nodules is reviewed by Sahli et al.. The limitations of current genetic markers, their cost-effectiveness and the next generation of tests currently being evaluated are discussed.

Another common ailment associated with aging is osteopenia, which leads to the high prevalence of fractures seen in the elderly population. The challenges of identifying individuals at risk of fractures and the uses and limitations of current therapies for osteopenia are discussed by Ramchand and Seeman. The relative merits of antiresorptive and anabolic therapies are discussed as are the alternative strategies of combining these therapies or using them sequentially.

The increasing speed of technological advances provides endocrinologists with ever more powerful tools for investigation, diagnosis and treatment. As the pressures of modern lifestyles involve major changes in how we live and eat and demographics markedly increase the elderly population the challenges endocrinologists face in the clinic are constantly evolving. This collection of articles illustrates the variety of these challenges across the different specialties within endocrinology and the dynamic nature of modern endocrinology.

## Author Contributions

Both authors have made a substantial, direct and intellectual contribution to the work and approved it for publication.

### Conflict of Interest

The authors declare that the research was conducted in the absence of any commercial or financial relationships that could be construed as a potential conflict of interest.

## References

[1] Tremmel M Gerdtham UG Nilsson PM Saha S. Economic burden of obesity: a systematic literature review. Int J Environ Res Public Health. (2017) 14:E435. 10.3390/ijerph14040435 28422077 PMC5409636

[2] Samaras K Tevaearai H Goldman M le Coutre J Holly JMP. With obesity becoming the new normal, what should we do? Front Endocrinol. (2019) 10:250. 10.3389/fendo.2019.00250 31057486 PMC6481296

[3] Kershaw EE Flier JS. Adipose tissue as an endocrine organ. J Clin Endocrinol Metab. (2004) 89:2548–56. 10.1210/jc.2004-0395 15181022

[4] Virtanen KA Lidell ME Orava J Heglind M Westergren R Niemi T. Functional brown adipose tissue in healthy adults. N Engl J Med. (2009) 360:1518–25. 10.1056/NEJMoa0808949 19357407

[5] Ahima RS Antwi DA. Brain regulation of appetite and satiety. Endocrinol Metab Clin North Am. (2008) 37:811–23. 10.1016/j.ecl.2008.08.005 19026933 PMC2710609

